# Long-term survival with sebelipase alfa enzyme replacement therapy in infants with rapidly progressive lysosomal acid lipase deficiency: final results from 2 open-label studies

**DOI:** 10.1186/s13023-020-01577-4

**Published:** 2021-01-06

**Authors:** Suresh Vijay, Anais Brassier, Arunabha Ghosh, Simona Fecarotta, Florian Abel, Sachin Marulkar, Simon A. Jones

**Affiliations:** 1 Birmingham Children’s Hospital NHS Foundation Trust, Steelhouse Lane, Birmingham, B4 6NH UK; 2 Hospital Necker Enfants Malades, APHP, Paris, France; 3 Manchester Centre for Genomic Medicine, Manchester University NHS Foundation Trust, St. Mary’s Hospital, Manchester, UK; 4 Federico II University, Naples, Italy; 5 Alexion Pharmaceuticals, Inc., Boston, MA USA

**Keywords:** Dyslipidemias, Growth, Wolman disease, Lysosomal storage diseases, Transaminases

## Abstract

**Background:**

If symptomatic in infants, the autosomal recessive disease lysosomal acid lipase deficiency (LAL-D; sometimes called Wolman disease or LAL-D/Wolman phenotype) is characterized by complete loss of LAL enzyme activity. This very rare, rapidly progressive form of LAL-D results in severe manifestations leading to failure to thrive and death, usually by 6 months of age. We report results from 2 open-label studies of enzyme replacement therapy with sebelipase alfa, a recombinant human LAL, in infants with LAL-D: the phase 2/3 Survival of LAL-D Infants Treated With Sebelipase Alfa (VITAL) study (NCT01371825) and a phase 2 dose-escalation study (LAL-CL08 [CL08]; NCT02193867). In both, infants received once-weekly intravenous infusions of sebelipase alfa.

**Results:**

The analysis population contained 19 patients (9 in VITAL; 10 in CL08). Kaplan–Meier estimates of survival to 12 months and 5 years of age were 79% and 68%, respectively, in the combined population, and the median age of surviving patients was 5.2 years in VITAL and 3.2 years in CL08. In both studies, median weight-for-age, length-for-age, and mid-upper arm circumference-for-age *z* scores increased from baseline to end of study. Decreases in median liver and spleen volume over time were noted in both studies. Short-term transfusion-free hemoglobin normalization was achieved by 100% of patients eligible for assessment in VITAL, in an estimated median (95% confidence interval [CI]) time of 4.6 (0.3–16.6) months. In CL08, short-term transfusion-free hemoglobin normalization was achieved by 70% of patients eligible for assessment, in an estimated median (95% CI) time of 5.5 (3.7–19.6) months. No patient discontinued treatment because of treatment-emergent adverse events. Most infusion-associated reactions (94% in VITAL and 88% in CL08) were mild or moderate in severity.

**Conclusions:**

The findings of these 2 studies of infants with rapidly progressive LAL-D demonstrated that enzyme replacement therapy with sebelipase alfa prolonged survival with normal psychomotor development, improved growth, hematologic parameters, and liver parameters, and was generally well tolerated, with an acceptable safety profile.

## Background

Lysosomal acid lipase deficiency (LAL-D) is a rare, progressive, autosomal recessive disease caused by *LIPA* mutations [1–3]. It is characterized by minimal residual LAL activity in children and adults [1], which leads to a severely impaired ability to hydrolyze cholesteryl esters and triglycerides [3, 4], and by a complete absence of LAL activity in infants [1], which causes a more rapidly progressive, fatal disease course very early in life [1, 3].

The intracellular accumulation of lipids causes numerous disease manifestations [1, 3, 5]. In children and adults with LAL-D, liver involvement and dyslipidemia similar to heterozygous familial hypercholesterolemia dominate the clinical picture [1, 2]. Liver effects include hepatomegaly, liver dysfunction, and hepatic failure [1, 4]. In contrast, infantile-onset LAL-D (historically called Wolman disease or LAL-D/Wolman phenotype), a very rare form of the disease, is the most rapidly progressive disease presentation, and is usually fatal within the first 6 months of life [1, 6–8]. The predominant clinical features of infantile-onset LAL-D are early growth failure, severe hepatic disease—as evidenced by liver enlargement, elevation of transaminases, hyperbilirubinemia, coagulopathy, and hypoalbuminemia—and a macrophage activation syndrome, which are key contributors to early mortality [9–11]. Malabsorption is common in infants with LAL-D [1]. Infants are typically hospitalized within the first 2 to 3 months of life due to diarrhea, persistent vomiting, feeding difficulty, and growth failure [12].

Sebelipase alfa (Kanuma^®^, Alexion Pharmaceuticals, Inc.), the only approved treatment for LAL-D, is a recombinant human LAL enzyme replacement therapy indicated for the treatment of patients with a diagnosis of LAL-D [13]. Infants treated with sebelipase alfa have shown prolonged survival compared with historical controls [8, 9]. Interim results from a phase 2/3 study (Survival of LAL-D Infants Treated With Sebelipase Alfa [VITAL]; also known as LAL-CL03) of 9 infants with rapidly progressive LAL-D treated with sebelipase alfa, the final results of which are reported herein, showed that 6 (67%) survived to 12 months and 5 (56%) survived to 24 months [9]. Among 35 historical control patients, the median survival time was 3.7 months; 4 patients survived to at least 12 months, and 3 of those patients underwent hematopoietic stem cell transplantation (HSCT), including 1 who also received a liver transplant [8]. The Kaplan–Meier estimate of survival past age 12 months was 11% in the overall control population; in contrast, among the subset of 21 untreated patients with early growth failure, survival at 12 months was 0%.

We report final results from 2 studies (VITAL and LAL-CL08 [subsequently referred to as CL08]) that evaluated the safety and efficacy of sebelipase alfa in infants with rapidly progressive LAL-D.

## Results

### Patients

The VITAL study enrolled patients from May 2011 through December 2013 at 8 sites in 6 countries (United Kingdom [UK], United States [US], France, Ireland, Egypt, and Turkey). The CL08 study enrolled patients from June 2014 to May 2016 at 5 sites in 4 countries (US, Finland, and UK; 1 patient who initiated treatment in the UK subsequently transferred to a site in Italy to receive treatment). Nineteen patients were enrolled (10 males, 9 females) and received treatment in the 2 studies (VITAL, N = 9; CL08, N = 10); 13 patients completed the studies or switched to the commercial product upon study termination (CL08 only) (VITAL, n = 5; CL08, n = 8). Six patients died during the studies (4 in VITAL and 2 in CL08); none of the deaths was considered to be related to sebelipase alfa treatment. Baseline demographic and disease characteristics of the treated patients are summarized in Table 1. The CL08 population appeared to have more severe disease at baseline, based on lower World Health Organization (WHO) percentiles for weight-for-age and mid-upper arm circumference-for-age.

**Table 1:** Baseline patient demographic and disease characteristics (full analysis set)

Parameter	VITAL (N = 9)	CL08 (N = 10)
Age at first dose, months, median (range)	3.0 (1–6)	2.8 (0.5–4)
Sex, n (%)		
Male	5 (56)	5 (50)
Female	4 (44)	5 (50)
Race, n (%)		
American Indian or Alaska Native	0	1 (10)
Asian	1 (11)	6 (60)
Black	1 (11)	0
White	4 (44)	1 (10)
Other	0	2 (20)
Unknown	3 (33)	0
Anthropometric parameters, median (IQR)		
Weight-for-age, WHO percentile^a^	3.1 (1–8)^b^	1.1 (0.0–8)
Length (height)-for-age, WHO percentile^a^	1.8 (0.1–39)^b^	2.9 (0.6–50)^c^
Mid-upper arm circumference-for-age, WHO percentile^a^	0.01 (0.0–0.3)^d^	0.001 (0.0–0.3)^e^
Liver parameters, median (range)		
ALT		
U/L	145.0 (16–297)	37.0 (28–248)^c^
µkat/L	2.42 (0.3–5.0)	0.62 (0.5–4.1)^c^
AST		
U/L	125.0 (71–716)	99.5 (56–441)^b^
µkat/L	2.09 (1.2–12.0)	1.66 (0.9–7.4)^b^
Ferritin		
µg/L (ng/mL)	586.3 (253–48,740)^f^	1750.5 (481–3020)^g^
Albumin, g/L	29.0 (13–40)	20.0 (18–29)^c^
Total bilirubin		
mg/dL	1.7 (0.2–27)	0.7 (0.2–3)
µmol/L	29.0 (3–464)^b^	12.0 (4–52)^c^
Hematologic parameters, median (range)		
Hemoglobin, g/L	93.0 (1–103)	90.0 (81–131)^f^
Platelets, 10^9^/L	173.0 (3–563)	146.0 (56–235)^f^
LAL-D manifestations, n (%)		
Hepatomegaly and/or splenomegaly	9 (100)	9 (90)
Diarrhea and/or vomiting	9 (100)	9 (90)
Adrenal calcification	9 (100)	5 (50)
Failure to thrive/malnutrition	9 (100)	5 (50)
Liver volume, MN, median (range)	3.4 (3–4)^h^	3.2 (0.1–4)^f^
Spleen volume, MN, median (range)	7.0 (3–10)^g^	5.8 (0.7–15)^b^

*ALT* alanine aminotransferase, *AST* aspartate aminotransferase, *CDC* Centers for Disease Control and Prevention, *IQR* interquartile range, *LAL-D* lysosomal acid lipase deficiency, *MN* multiples of normal, *WHO* World Health Organization

^a^Based on WHO Multicenter Growth Reference Study Group, 2006 and 2007 WHO Child Growth Standards [18, 19]. After age 2, standardization is based on 2000 CDC Growth Chart [20]

^b^n = 8

^c^n = 9

^d^n = 4

^e^n = 5

^f^n = 7

^g^n = 2

^h^n = 3

### Efficacy

#### Survival

**Fig. 1:**
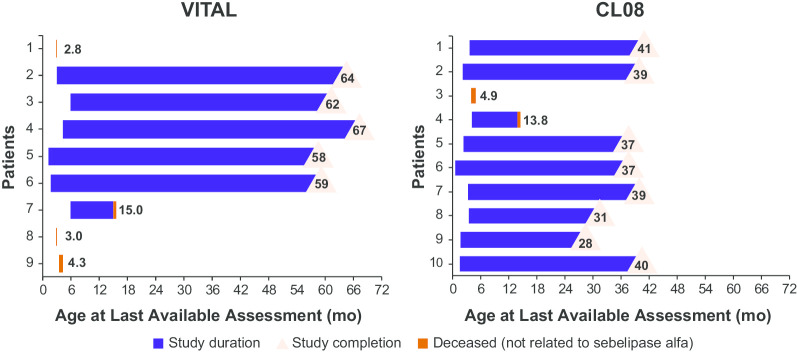
Patient Survival at End of Study

**Fig. 2:**
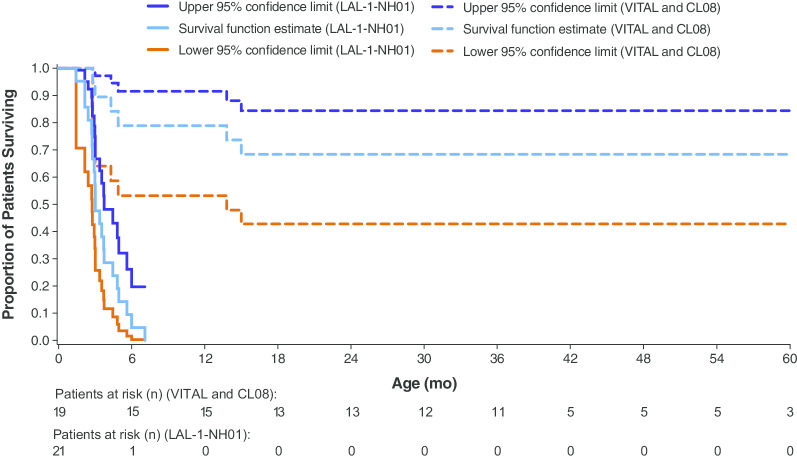
Kaplan–Meier Estimate of Survival, Birth to 60 Months. Kaplan–Meier estimates in VITAL and CL08 study participants compared with untreated infants with growth failure due to rapidly progressive LAL-D (natural history study LAL-1-NH01) [9]. The patient population in LAL-1-NH01 was similar in clinical characteristics to VITAL and CL08 patient populations. Adapted with permission from *Orphanet Journal of Rare Diseases* (Jones et al. [9]). LAL-D, lysosomal acid lipase deficiency

Patient survival in each study is shown in Fig. 1. The median (range) age of surviving patients at end of study was 5.2 (4.8–5.6) years in VITAL and 3.2 (2.3–3.4) years in CL08. In VITAL, the Kaplan–Meier estimate of survival to 12 months of age was 67%, and to 4 years was 56%. In CL08, the Kaplan–Meier estimate of survival to 12 months of age was 90%, and to 3 years was 80%. In the combined population, the Kaplan–Meier estimate of survival to 12 months of age was 79%, and to 5 years was 68%. After excluding the 4 patients who died after receiving 4 or fewer infusions of sebelipase alfa, the Kaplan–Meier estimate of survival to 12 months of age was 100% and to 5 years was 87% in the combined population. In Fig. 2, the survival analysis for the combined population is compared with a similar analysis of 12-month data from untreated infants with growth failure due to rapidly progressive LAL-D (natural history study LAL-1-NH01), in which none of the untreated infants survived past 12 months of age [9].

#### Growth and functional development

**Fig. 3:**
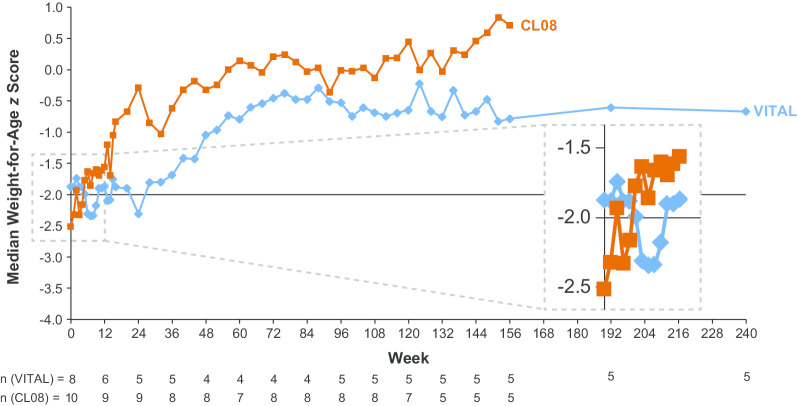
Median Weight-for-Age *z* Scores. Data reported weekly through week 16, then every 4 weeks through week 156 in both studies, then at week 192 (month 48) and week 240 (month 60) in VITAL only. The horizontal line indicates the threshold for underweight [14]

**Fig. 4:**
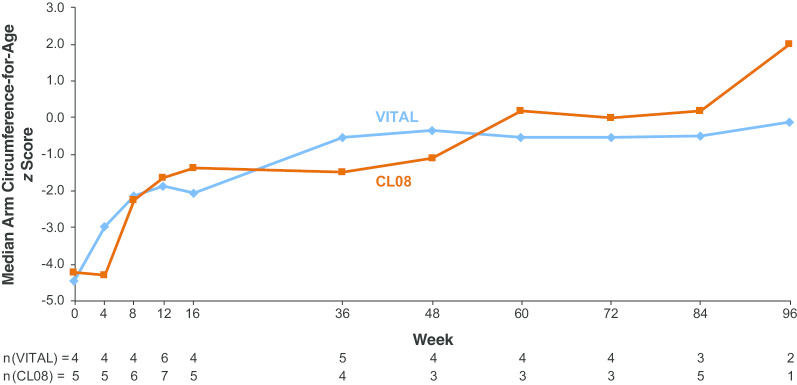
Median Mid-Upper Arm Circumference-for-Age *z* Scores. Data reported at weeks 0, 4, 8, 12, 16 in VITAL and CL08, at weeks 20 and 24 in VITAL only, and then every 12 weeks from week 36 through week 96 in both studies; values are shown only for the weeks at which data are available from both studies

Median weight-for-age *z* scores in VITAL and CL08 are shown in Fig. 3; the horizontal line at − 2 standard deviations indicates the threshold for underweight established by the United Nations Children’s Fund [14]. The median (range) *z* score increased from − 1.875 (− 4.79 to 0.74; n = 8) at baseline to − 0.669 (− 1.41 to 1.87; n = 5) at week 240 (month 60; last visit with data available for > 4 patients) in VITAL, and from − 2.515 (− 4.45 to 0.84; n = 10) at baseline to 0.711 (− 0.51 to 1.08; n = 5) at week 156 in CL08. The median (range) length-for-age *z* score increased from − 2.290 (− 3.91 to 0.87; n = 8) at baseline to − 0.386 (− 1.90 to 1.76; n = 5) at week 240 (month 60; last visit with data available for > 2 patients) in VITAL, and from − 1.900 (− 3.20 to 0.47; n = 9) at baseline to 0.209 (− 1.20 to 0.73; n = 5) at week 156 in CL08. Median mid-upper arm circumference-for-age *z* scores in VITAL and CL08 are shown in Fig. 4. The median (range) mid-upper arm circumference-for-age *z* score increased from − 4.450 (− 5.98 to –2.50; n = 4) at baseline to − 0.490 (− 1.96 to 0.43; n = 3) at week 84 (month 21; last visit with data available for > 2 patients) in VITAL, and from − 4.200 (− 5.88 to –1.73; n = 5) at baseline to 0.190 (− 1.42 to 1.08; n = 5) at week 84 (last visit with data available for > 2 patients) in CL08.

In both studies, patients remained generally stable in all 4 skill areas of the Denver II Developmental Screening Test (language, fine motor–adaptive, gross motor, personal-social) through end of study. In VITAL, none of the surviving patients was untestable on a postdose assessment, and no patient treated for at least 24 weeks tested as “abnormal” in any skill area at any time point. In CL08, no patient treated for at least 24 weeks was untestable in any skill area at any subsequent time point.

#### Liver, hematologic, and lipid effects

**Fig. 5:**
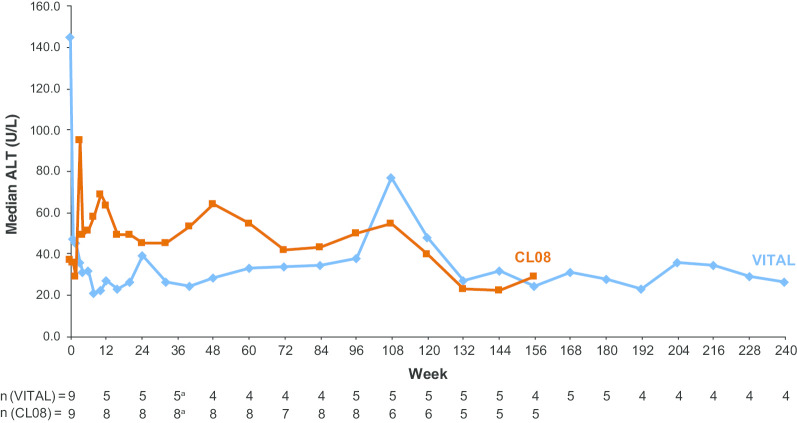
Median ALT Levels. To convert to SI units (μkat/L), multiply by 0.0167. ALT, alanine aminotransferase. ^a^  Patient n numbers are for weeks 32 and 40

Liver function, hematologic measures, and lipid levels at baseline and end of study are shown in Table 2. Median alanine aminotransferase (ALT) levels over time in VITAL and CL08 are illustrated in Fig. 5. In VITAL, among the 6 patients who survived beyond week 4, 4 had abnormal ALT levels at baseline, and all 4 experienced normalization of ALT during sebelipase alfa treatment. Similarly, 4 of 6 patients who survived beyond week 4 had abnormal aspartate aminotransferase (AST) levels at baseline, and all 4 experienced normalization of AST during treatment. In CL08, normalization of ALT was achieved for the 3 patients with abnormal baseline ALT levels, and normalization of AST was achieved for 50% of patients with abnormal baseline AST levels. As shown in Table 2, decreases in median liver and spleen volume over time were noted in both studies.

**Table 2:** Liver, hematologic, and lipid effects

Parameter	VITAL (N = 9)		CL08 (N = 10)	
	Baseline (n = 9), median (range)	End of study^a^ (n = 4), median (range)	Baseline (n = 9), median (range)	End of study^b^ (n = 5), median (range)
ALT				
U/L	145.0 (16–297)	26.5 (18–38)	37.0 (28–248)	29.0 (22–106)
µkat/L	2.42 (0.3–5.0)	0.44 (0.3–0.6)	0.62 (0.5–4.1)	0.48 (0.4–1.8)
AST				
U/L	125.0 (71–716)	44.5 (41–54)	99.5 (56–441)^c^	44.0 (38–110)
µkat/L	2.09 (1.2–12.0)	0.74 (0.7–0.9)	1.66 (0.9–7.4)^c^	0.73 (0.6–1.8)
Ferritin				
µg/L (ng/mL)	586.3 (253–48,740)^d^	93.5 (42–123)	1750.5 (481–3020)^e^	62.1 (49–75)
Albumin, g/L	29.0 (13–40)	32.0 (27–37)	20.0 (18–29)	33.0 (20–37)^f^
Hemoglobin, g/L	93.0 (1–103)	115.5 (99–123)	90.0 (81–131)^d^	113.0 (103–129)^f^
Total cholesterol				
mg/dL	139.2 (67–225)^g^	112.1 (93–131)^h^	125.7 (97–1063)^i^	106.3 (85–205)^f^
mmol/L	3.6 (2–6)^g^	2.9 (2–3)^h^	3.3 (3–28)^i^	2.8 (2–5)^f^
LDL-C				
mg/dL	109.4 (19–194)^g^	64.2 (63–75)^h^	119.0 (62–143)^h^	76.6 (53–137)^h^
mmol/L	2.8 (0.5–5)^g^	1.7 (2–2)^h^	3.1 (2–4)^h^	2.0 (1–4)^h^
HDL-C				
mg/dL	8.9 (0–10)^g^	18.9 (13–19)^h^	9.4 (8–12)^f^	13.1 (13–29)^h^
mmol/L	0.2 (0.0–0.3)^g^	0.5 (0.3–0.5)^h^	0.2 (0.2–0.3)^f^	0.3 (0.3–0.8)^h^
Triglycerides				
mg/dL	163.9 (31–218)^g^	99.2 (90–237)^h^	265.7 (71–424)^g^	151.1 (133–195)^h^
mmol/L	1.9 (0.4–3)^g^	1.1 (1–3)^h^	3.0 (0.8–5)^g^	1.7 (2–2)^h^
Liver volume, MN	3.4 (3–4)^h^	1.6 (0.3–3)^h,j^	3.2 (0.1–4)^d^	1.9 (1–2)^e^
Spleen volume, MN	7.0 (3–11)^e^	2.6 (2–7)^h,j^	5.8 (0.7–15)^c^	4.0 (2–6)^e^

*ALT* alanine aminotransferase, *AST* aspartate aminotransferase, *HDL-C* high-density lipoprotein cholesterol; LDL-C, low-density lipoprotein cholesterol; MN, multiples of normal

^a^Week 240 (month 60; last visit with n > 2)

^b^Week 156 (month 39)

^c^n = 8

^d^n = 7

^e^n = 2

^f^n = 4

^g^n = 5

^h^n = 3

^i^n = 6

^j^Week 120 (month 30; last visit with n > 1)

Liver biopsy was not obtained at baseline in VITAL or CL08 due to the critically ill status of the infants and the potential risks associated with the procedure. In VITAL, 1 patient underwent a liver biopsy (and a lymph node biopsy) on day 578 for abdominal adenomegalies and suspicion of malignancy; pathology results relating to LAL-D were not reported. No other patients in VITAL underwent liver biopsy. In CL08, liver histopathology data were available for 6 patients. At the first assessment (on or after week 48), patients generally had relatively little fibrosis (as indicated by Ishak score), microvesicular steatosis, and lobular or portal inflammation, and low scores for percentages of collagen, macrophages, and fibrogenic cells. Four patients in the study had repeated assessments, with the first assessment occurring at either week 48 (3 patients) or week 63 (1 patient) and follow-up assessments at week 96 (3 patients), week 119 (1 patient), and/or week 152 (1 patient). A review of the changes in liver histopathology in these 4 patients did not suggest any consistent trends over time.

Marked decreases in median serum ferritin levels occurred from baseline to end of study in both studies and median albumin levels and median hemoglobin levels increased in both studies (Table 2). Depending on the clinical status, patients received transfusions or blood products as part of their clinical care.

Transfusion-free hemoglobin normalization (TFHN) of at least 4 weeks (short-term TFHN)—which meant that hemoglobin levels were consistently above the age-adjusted lower limit of normal over a minimum period of 4 weeks, with no transfusions during this period or for 2 weeks prior to the first hemoglobin measurement in the period—was achieved by the 6 (100%) patients in VITAL who were eligible for assessment, which included the 3 patients who had low hemoglobin levels at baseline. The estimated median (95% confidence interval [CI]) time to achieve TFHN was 4.6 (0.3–16.6) months. Two of these patients also achieved TFHN maintenance of 13 weeks or more (sustained early TFHN). In CL08, short-term TFHN was achieved by 7 (70%) patients, with an estimated median (95% CI) time to achieve TFHN of 5.5 (3.7–19.6) months. Most patients maintained TFHN for at least 13 weeks; however, no patient met the formal criterion for TFHN maintenance, because TFHN was not attained until after week 8.

Median (range) levels of total cholesterol, low-density lipoprotein cholesterol, high-density lipoprotein cholesterol, and triglycerides are summarized in Table 2; in general, lipid data were more limited, but available data suggest trends toward improvement in the serum lipid profile of patients in both studies. In VITAL, 4 patients received lipid-modifying agents (cholestyramine, n = 3; other, n = 1). Of these 4 patients, 3 were on a lipid-modifying agent at the start of the study and continued receiving it throughout the study at the same dose or a higher dose; the fourth patient started a lipid-modifying agent during the study and the dose was increased during the study. In CL08, lipid-modifying medication (fenofibrate 145 mg once daily) was routinely administered to 1 patient throughout the entire study. This patient also received 2 potentially lipid-modifying agents (*Carthamus tinctorius* oil and omega-3-acid ethyl ester) from day 651 through end of study; 2 other patients received treatment with transient courses of lipid-modifying agents (atorvastatin [a 13-day course] or cholestyramine [several brief courses]).

#### Nutritional status

Treatment with sebelipase alfa was associated with a trend toward normalization of diet, although most patients still required some form of nutritional modification (eg, medium-chain triglyceride formula, fat-restricted diet, or fat-soluble vitamin supplementation). In the combined studies, 9 of 13 surviving patients required parenteral nutrition at some point during the study, typically at initiation of therapy or during intercurrent illness. In both studies, frequent adjustments were made to meet the nutritional needs of each patient during treatment.

### Safety

A total of 1249 infusions were administered in VITAL, and 1193 infusions were administered in CL08. Treatment-emergent adverse events (TEAEs) were reported for all 19 patients in the 2 studies (Table 3). No patient discontinued treatment because of TEAEs. Ninety-five percent of TEAEs in VITAL and 98% in CL08 were mild or moderate in severity; 4 patients (44%) in VITAL and 7 patients (70%) in CL08 experienced 1 or more severe TEAEs. In VITAL, reporting of TEAEs by severity included 4 patients who died and 1 who had life-threatening TEAEs (diarrhea, dehydration, and metabolic acidosis; all considered serious, unrelated to sebelipase alfa, and recovered/resolved). The most frequently reported TEAEs that were considered related or possibly related to treatment (occurring in ≥ 30% of patients) were: vomiting, pyrexia, and urticaria in VITAL, and tachycardia, vomiting, pyrexia, irritability, agitation, respiratory distress, tachypnea, and urticaria in CL08. Serious adverse events (SAEs) were reported in all 19 patients. Serious adverse events related or possibly related to study drug were reported in 6 patients, 1 (11%) in VITAL and 5 (50%) in CL08; with the exception of 1 patient in CL08, these were infusion-associated reactions (IARs). The patient in CL08 who had a non-IAR SAE had initiated sebelipase alfa treatment at 2 weeks of age and developed hypertension and moderate proteinuria at 6 months of age, along with high anti-drug antibody (ADA) titers. The patient received treatment with amlodipine, rituximab, and bortezomib, and underwent HSCT at 30 months. Six months after HSCT, the patient developed nephrotic syndrome, suspected to have resulted from the deposition of circulating soluble immune complexes in the glomerulus, and received multiple albumin infusions, furosemide, prednisolone, lisinopril, and bortezomib. The patient’s proteinuria had almost normalized by 8 months [15].

**Table 3:** Summary of adverse events (full analysis set)

Event	VITAL (N = 9), n (%)	CL08 (N = 10), n (%)
Any TEAE	9 (100)	10 (100)
Mild or moderate TEAEs	0 (0)	3 (30)
Any treatment-related or possibly related TEAE	6 (67)	8 (80)
Any SAE	9 (100)	10 (100)
Any treatment-related or possibly related SAE	1 (11)	5 (50)
Any IAR	5 (56)	8 (80)
Mild or moderate IARs	5 (56)	6 (60)
Dose modification due to a TEAE^a^	7 (78)	7 (70)
Discontinuation due to a TEAE	0 (0)	0 (0)
Death	4 (44)	2 (20)

*IAR* infusion-associated reaction, *SAE* serious adverse event, *TEAE* treatment-emergent adverse event

^a^Includes dose increases, dose decreases, drug interruptions, and study drug withdrawal

Infusion-associated reactions were reported in 13 of 19 patients. Of the 54 IARs reported in VITAL, 94% were mild or moderate in severity, and of the 98 IARs reported in CL08, 88% were mild or moderate in severity. The most frequently reported IARs in VITAL were pyrexia (33%), urticaria (33%), vomiting (33%), tachycardia (22%), and pallor (22%); in CL08, they were tachycardia (70%), pyrexia (60%), irritability (50%), agitation (40%), and urticaria (40%). One patient in CL08 experienced IARs that required a per-protocol desensitization procedure involving administration of lower concentration solutions of sebelipase alfa over prolonged infusion times, with stepwise increases in infusion rate and an increase in dose from 0.35 mg/kg once weekly (qw) to the full planned dose of 3.0 mg/kg qw over the initial 4 infusions of the desensitization period [16]. In all other patients IARs were successfully managed and resolved using local regular treatment protocols, as in studies of other enzyme replacement therapies [17].

As noted previously, 6 patients died during the studies; none of the deaths was considered to be related to sebelipase alfa treatment. In VITAL, 3 of the 4 patients died after receiving up to 4 doses of sebelipase alfa (1 patient died at 2.8 months of age due to liver failure as a result of LAL-D; 1 patient died at 3.0 months of age due to peritoneal hemorrhage following a non–study-related procedure [abdominal paracentesis]; and 1 patient died at 4.3 months of age due to cardiac arrest). One patient in VITAL had sudden cardiac death at 15.0 months of age. In CL08, 1 patient died at 4.9 months of age from pericardial effusion due to necrosis of the atrial wall associated with leakage from an indwelling intravenous (IV) line, and 1 patient died at 13.8 months of age from septicemia.

Table 4 summarizes the final doses of sebelipase alfa among surviving patients at the end of the VITAL and CL08 studies and immunogenicity results during the studies. One patient in VITAL switched to an every-other-week (qow) dosing regimen beginning at week 122, but ultimately reverted back to a qw dosing regimen beginning at week 155 due to elevated serum transaminase levels during qow dosing; serum transaminases returned to within the normal range with qw dosing. Three additional patients in VITAL had isolated instances of qow dosing during the study, including 1 patient who was intermittently hospitalized due to intercurrent adenovirus and rotavirus infections. No patient in CL08 switched to a qow regimen.

**Table 4:** Sebelipase alfa dosing status and immunogenicity

	VITAL (N = 9)	CL08 (N = 10)
Final dose, surviving patients, n/N	5/9	8/10
1.0 mg/kg qw, n	0	1^a^
3.0 mg/kg qw, n	3	3
5.0 mg/kg qw, n	2	4
Week of first 3.0 mg/kg dose, median (range)^b^	14 (6–91)	12 (4–58)
Immunogenicity during the study^c^		
Anti-drug antibody test, n positive/N tested	4/7	6/10
Neutralizing assay, n positive/N tested	3/4	6/6
Inhibit cellular uptake of LAL	3/4	6/6
Inhibit LAL enzyme activity	2/4^d^	6/6

*LAL* lysosomal acid lipase, *qw* once weekly

^a^One patient received successive dose escalations up to a maximum dose of 7.5 mg/kg qw. This patient had subsequent successive dose reductions to 1.0 mg/kg qw after a successfully engrafted bone marrow transplant at week 101

^b^Among surviving patients

^c^Among all patients with posttreatment assessments

^d^These patients also had neutralizing antibodies that inhibit cellular uptake of LAL

In VITAL, among 7 patients with posttreatment immunogenicity data, 4 (57%) were positive for ADAs during at least 1 assessment. Anti-drug antibody positivity was first detected at week 5 for 1 patient, week 8 for 2 patients, and week 59 for 1 patient, and ADA positivity persisted (> 1 ADA-positive result) in all 4 patients. One patient remained ADA-positive at the majority of assessments from the initial ADA-positive result at week 5 through the end of the study, 2 patients were ADA-positive at the majority of assessments for a period of 110 or 208 weeks but then tested ADA-negative for the remainder of the study, and 1 patient had only intermittent low-titer ADAs interspersed with periods during which results were ADA-negative. Among the 4 ADA-positive patients, peak titers ranged from 223 to 4721; these were reported at the follow-up visit in the patient with intermittent low-titer ADAs, and between weeks 35 and 216 in the other 3 patients. Those 3 patients had titers that decreased during continued treatment with sebelipase alfa; 2 of these 3 patients tested ADA-negative at multiple assessments at the end of the study. Three of the 4 ADA-positive patients tested positive for neutralizing antibodies that inhibit the cellular uptake of LAL, 2 of whom also tested positive for antibodies that inhibit LAL enzyme activity.

In CL08, 6 patients developed ADAs during treatment, with positivity first detected at week 5 for 1 patient, week 8 for 2 patients, and weeks 12, 20, and 60 (1 patient each). Two of these patients tested ADA-positive at their first postdose assessment (at week 8 and week 12), and therefore may have developed ADAs prior to this time point. The 4 patients who tested ADA-positive at or prior to week 12 all had high antibody titers, with peak titers ranging from 46,774 to 302,963 between weeks 86 and 118; the patient who tested ADA-positive at week 20 had a moderate antibody titer that peaked at 2958 at week 84 and fluctuated but showed a decreasing trend through the end of the study, and the patient who had tested ADA-positive at week 60 continued to have low-titer ADAs throughout treatment with a peak titer of 292 at the follow-up visit. All 6 ADA-positive patients tested positive for neutralizing antibodies that inhibited both LAL enzyme activity and cellular uptake. Data suggested that neutralizing antibodies had an impact on clinical response in 3 patients. These 3 patients had persistent and notably higher ADA titers (peak titers ranging from 222,070 to 302,963) than the ADA-positive patients in whom sebelipase alfa efficacy was not affected (peak titers ranging from 292 to 46,774). The 3 unrelated patients in whom sebelipase alfa efficacy was affected by ADAs had a complete deletion of *LIPA*, including the neighboring gene for cholesterol 25-hydroxylase, *CH25H* (Table 5). In these 3 patients, the development of high ADA titers was associated with decreased weight for age and other parameters of failure to thrive, suggestive of decreased clinical efficacy. Loss of efficacy resulted in sebelipase alfa dose increases (all 3 patients) and other clinical measures, such as bone marrow transplant (week 101) and immunomodulatory therapy with bortezomib (week 108) (n = 1) and immunomodulatory therapy and immune supplementation therapy with IV immunoglobulin, rituximab, and bortezomib (intermittently between weeks 85 and 128), followed by HSCT and bortezomib (week 131) (n = 1). After undergoing bone marrow transplant or HSCT, these patients were able to re-establish clinical efficacy at a reduced dose of sebelipase alfa, which was associated with a decrease in the ADA titer. There was no clear relationship between ADA status and the risk of IARs.

**Table 5:** *LIPA* genotype (assessed by central laboratory)

Study	Patient #	Allelic mutations	Effects of mutation	Variant severity
VITAL				
	1	ND	NA	NA
	2	c.46A > C HOM C c.676-42G > A HET c.966 + 46C > T HET c.894G > A HET c.455 T > C HET	p.Thr16Pro rs1051338 Intronic rs1556478 Intronic rs3802656 p.Gln298Gln rs116928232 p.Leul52Pro	Common variant Common variant Common variant Documented pathogenic VUS
	3	c.884A > G HET	p.His295Arg	VUS
	4	c.539-5C > T HET c.482delA HET c.538G > A HET	Intronic rs2297472 p.Asn161Ilefs*19 p.Gly180Ser	Common variant Documented pathogenic VUS
	5	c.539-5C > T HET c.676-42G > A HOM c.966 + 46C > T HOM c.193C > T HET c.894G > A HET c.419G > C HOM	Intronic rs2297472 Intronic rs1556478 Intronic rs3802656 p.Arg65Stop p.Gln298Gln rs116928232 p.Trp140Ser	Common variant Common variant Common variant Documented pathogenic Documented pathogenic VUS
	6	c.676-2A > G HOM	Intronic	Documented pathogenic
	7	c.350_351insCC HET c.797G > T HET	p.Met117IlefsStop45 p.Gly266Val	Expected Pathogenic VUS
	8	ND	NA	NA
	9	c.67G > A HOM c.539-5C > T HET c.260G > T HOM	p.Gly23Arg rs1051339 Intronic rs2297472 p.Gly87Val	Common variant Common variant Documented pathogenic
CL08				
	1	c.594dupT HOM	p.Ala199Cysfs*13	Documented pathogenic
	2^a,b^	c.67G > A HOM c.539-5C > T HOM	p.Gly23Arg Intronic	Common variant Common variant
	3	ND	NA	NA
	4	ND	NA	NA
	5^a^	ND	NA	NA
	6^a^	ND	NA	NA
	7	c.229G > T HOM	Intronic	Documented pathogenic
	8	ND	NA	NA
	9	c.46A > C HOM c.658C > T HOM c.539-5C > T HOM c.894 + 1G > A HOM	p.Thr16Pro p.Pro220Ser Intronic Splicing	Common variant VUS^c^ Common variant Documented pathogenic
	10	c.892C > T^d^	p.Gln298*	Documented pathogenic

*HET* heterozygous, *HOM* homozygous, *NA* not applicable, *ND* not done, *VUS* variant of unknown significance

^a^Patient was homozygous for whole *LIPA* deletion, including the neighboring gene for cholesterol 25-hydroxylase, *CH25H*, based on testing at a local laboratory

^b^Gene sequencing data from the central laboratory identified 2 polymorphisms, both common variants for the patient. It is recognized that the results from the central and local laboratories are discrepant, in that allelic variants should not have been detected in a patient who is homozygous for a whole *LIPA* deletion. Investigation into the cause of this discrepancy is ongoing

^c^Novel missense mutation

^d^The testing laboratory did not report whether the patient was heterozygous or homozygous for the given allelic mutation; however, both parents were heterozygous for this mutation

## Discussion

In these 2 open-label clinical studies of infants with rapidly progressive LAL-D, enzyme replacement therapy with sebelipase alfa qw for up to 5 years (VITAL) or up to 3 years (CL08) prolonged survival, with normal psychomotor development. In VITAL, the Kaplan–Meier estimate of survival to 12 months of age was 67% and to 4 years was 56%. In CL08, the estimates were 90% to 12 months of age and 80% to 3 years. For the combined studies, the Kaplan–Meier estimate of survival to 5 years was 68% in all patients and 87% in patients who received more than 4 infusions of sebelipase alfa. In contrast, untreated infantile-onset LAL-D is typically fatal by 6 months of age [1, 6, 7], and in a population of untreated infants with LAL-D with growth failure in a natural history study (LAL-1-NH01), none survived past 12 months of age [8]. At the end of the respective studies, the median age of surviving patients was 5.2 years in VITAL and 3.2 years in CL08.

Improvements in liver function and weight gain with sebelipase alfa treatment were sustained over time. While liver histopathology data did not show any consistent trends over time, the lack of baseline data and limited number of patients who underwent the optional liver biopsy procedure (n = 4 in CL08) preclude any firm conclusions. Possible reasons for the improved survival and weight gain observed in CL08 compared with VITAL may be related to an evolution in the understanding of disease management in the later CL08 study, leading to better nutritional management and earlier initiation, using a higher starting dose, and faster dose escalation of sebelipase alfa in CL08 compared with VITAL. The results suggest that starting treatment as early as possible may allow for the achievement of better outcomes. Comparison of baseline characteristics between VITAL and CL08 suggests there was a more severely affected population in CL08, with a lower weight for age and a smaller mid-upper arm circumference for age. Baseline mid-upper arm circumference data suggest more severe malnutrition than would be predicted by weight alone, which was potentially confounded by the presence of abdominal organomegaly and ascites. Although detailed nutritional data were not collected in these studies, information from the treating centers indicated that many patients still required a highly modified, low-fat diet. Achievement of optimal outcomes in this group of infants with complex disease requires a multidisciplinary approach to treatment and care, including early initiation and adequate dosing of sebelipase alfa along with a modified, fat-reduced diet.

Sebelipase alfa was generally well tolerated, consistent with its known safety profile [13]. No patient discontinued treatment because of TEAEs, and none of the 6 deaths that occurred during the study was considered to be related to treatment with sebelipase alfa. Of the IARs reported in VITAL and CL08, 94% and 88%, respectively, were mild or moderate. Desensitization was required for 1 patient in CL08. This involved administration of lower-concentration solutions of sebelipase alfa over prolonged infusion times, and ultimately led to the ability to administer the full planned dose, demonstrating that successful implementation of rapid desensitization is possible in affected patients [16]. The incidence of neutralizing antibodies was higher in CL08 (100% of patients with ADAs) than in VITAL (75% of patients with ADAs). In the combined studies, 8 of 10 patients tested positive for neutralizing antibodies that inhibited both LAL enzyme activity and cellular uptake, 1 patient tested positive for neutralizing antibodies that inhibited cellular uptake only, and 1 tested negative for neutralizing antibodies. In patients without whole *LIPA* deletion, ADA titers in the patient who tested positive for neutralizing antibodies that inhibited cellular uptake only (peak = 296) and the patient who tested negative for neutralizing antibodies (peak = 223) were generally much lower and less variable than in patients who tested positive for neutralizing antibodies that inhibited both LAL enzyme activity and cellular uptake (peak range = 292–46,774). The 3 patients with whole *LIPA* deletion had peak ADA titers exceeding 222,000. Although there was considerable fluctuation in ADA titers over time, there was a trend toward reduction with continued sebelipase alfa treatment. A total loss of LAL enzyme expression may increase the risk of immunogenicity, which could contribute to ADA development in some patients. Although there was no clear relationship between genotype and development of ADAs or occurrence of IARs in VITAL, CL08 contained patients with a more severe LAL-D genotype, including 3 patients with a complete deletion of *LIPA*, all of whom developed high-titer ADAs during treatment with sebelipase alfa and had diminished clinical efficacy over time.

These studies assembled and investigated an ultrarare disease patient population, had a long duration and follow-up, and included a comprehensive battery of clinical, laboratory, and developmental and social outcome measures. Their limitations include their open-label designs and small population sizes, which are consistent with a rare disease study and make it difficult to distinguish dose effects and do not allow for statistical comparisons. In addition, their lack of a placebo group required the use of a historical control population as a reference. It will be of interest to determine whether potential predictors of survival can be identified in future analyses of infants with rapidly progressive LAL-D.

## Conclusions

In 2 studies of infants with rapidly progressive LAL-D, sebelipase alfa enzyme replacement therapy prolonged survival, with normal psychomotor development in surviving patients as assessed by the Denver II Developmental Screening Test, improved growth and liver and lipid parameters, and was generally well tolerated, with an acceptable safety profile that was consistent with that seen in other sebelipase alfa clinical studies. The majority of surviving patients were ADA-positive during at least 1 assessment; however, an association with clinical efficacy and safety cannot yet be established based on these data. These findings support the long-term efficacy and safety of sebelipase alfa treatment in infants with rapidly progressive LAL-D and the importance of making a timely diagnosis to allow for early treatment.

## Methods

### Study designs

Both studies were open-label dose-escalation studies; VITAL was a phase 2/3 study, and CL08 was a phase 2 study (ClinicalTrials.gov registration numbers: NCT01371825 and NCT02193867, respectively). Eligible participants received qw IV infusions of sebelipase alfa for up to 3 years (CL08) or 5 years (VITAL). The primary objective of VITAL was to evaluate the effect of sebelipase alfa therapy on survival at 12 months of age in children with growth failure due to LAL-D, and that of CL08 was to evaluate the safety and tolerability of sebelipase alfa in infants with rapidly progressive LAL-D. A secondary objective was to evaluate the effect of sebelipase alfa therapy on survival at (CL08) and past 12 months of age (VITAL and CL08).

### Patients

Both studies required participants to have a laboratory-confirmed diagnosis of LAL-D (LAL activity or molecular testing). Key inclusion criteria for VITAL were growth failure or evidence of a rapidly progressive disease course with onset before 6 months of age; growth failure was defined by crossing below the 2nd percentile lines on standard weight-for-age curves over time, or below 10th percentile weight-for-age lines and falling from the curve, or loss of 5% or more of birth weight. For CL08, eligible patients were required to show signs or symptoms of rapid disease progression requiring urgent medical intervention, including, but not limited to: (1) marked abdominal distension and hepatomegaly; (2) failure to thrive, as evidenced by weight-for-height at least 2 standard deviations below the mean for sex and age; weight curve crossed downward by more than 2 major percentile lines on the WHO growth curves after a previous stable pattern of growth; (3) disturbance of coagulation; (4) severe anemia; (5) a sibling with a rapidly progressive course of LAL-D.

Patients were excluded from the studies if they had a clinically important concurrent disease or comorbidity or had received a previous hematopoietic stem cell or liver transplant. Patients were excluded from VITAL if they were more than 24 months of age at enrollment; patients more than 8 months of age on the date of the first infusion of sebelipase alfa were not eligible for the primary efficacy analysis (survival to 12 months of age). Patients were excluded from CL08 if they were more than 8 months of age at the time of the first dose of sebelipase alfa.

### Dosing

**Fig. 6:**
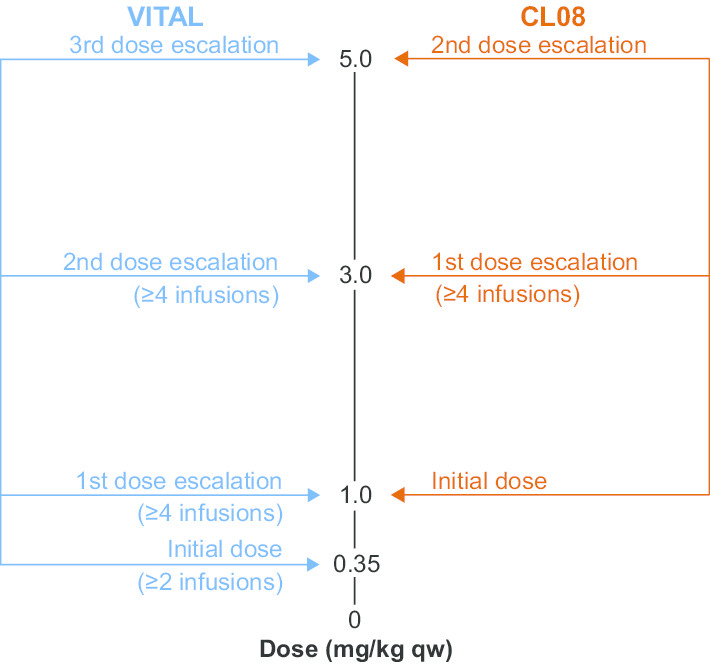
Dose Escalation in VITAL and CL08

Both studies permitted dose escalations of sebelipase alfa for eligible patients (Fig. 6). In VITAL, the starting dose was 0.35 mg/kg qw, with escalation to 1.0 mg/kg. If any patient met protocol-defined dose-escalation criteria (see the Additional file 1: Protocol-Defined Dose Escalation Criteria), escalation to 3.0 mg/kg was considered. Further escalation to 5.0 mg/kg was permitted at the discretion of the investigator and study sponsor in patients who continued to meet dose-escalation criteria [9]. In CL08, the initial dose was 1.0 mg/kg qw, with escalation to 3.0 mg/kg for patients who met protocol-defined dose-escalation criteria. Dose escalation to 5.0 mg/kg was considered for patients who continued to meet dose-escalation criteria. Under a country-specific protocol amendment, patients in the UK could be considered for a further dose escalation to 7.5 mg/kg qw if a thorough case review indicated that a patient continued to have evidence of disease progression at a dose of 5.0 mg/kg qw.

In both studies, patients who were on treatment for at least 96 weeks and who had been receiving a stable dose of sebelipase alfa for at least 24 weeks could be considered for a change in dosing schedule to qow infusions of sebelipase alfa. Such patients received the same dose per infusion that they had been receiving on their stable qw dosing schedule. Any subject receiving qow dosing who subsequently met criteria for dose escalation was to either revert to his/her stable qw dosing schedule or, if applicable, escalate from 1.0 mg/kg qow to 3.0 mg/kg qow. After dose escalation, dose reduction could be considered if a patient could not tolerate a higher dose.

### Efficacy assessments

The major efficacy assessments across both studies were the effect of treatment on survival, growth and functional development, liver, hematologic, and lipid parameters, and nutritional status. Growth assessments included weight-for-age, length-for age, and mid-upper arm circumference-for-age *z* scores relative to WHO child growth standards [18–20]. Functional development was assessed by the clinician at baseline and at various time points throughout the studies with the Denver II Developmental Screening Test, a standardized measure that assesses fine motor–adaptive, gross motor, personal-social, and language skills [21].

#### Liver, hematologic, and lipid parameters

Changes from baseline in serum ALT, AST, ferritin, and albumin levels were measured over time in both studies. Abdominal imaging with ultrasound was used to measure liver and spleen volumes over time, and imaging results were reviewed locally and centrally. Liver and spleen volumes were expressed in terms of multiples of normal, in which normal was defined as 2.5% and 0.2% of body weight, respectively.

During VITAL, the protocol was amended to include optional liver biopsies for evaluation of hepatic histology after a patient had completed at least 1 year of treatment, with consent from the patient’s parent or legal guardian and where local regulations permitted, and subject to discretionary approval from each center's institutional review board/independent ethics committee. Liver biopsy procedures were to be performed according to the local institutional practices by a qualified professional. All biopsies collected were to be centrally evaluated by a pathologist with the appropriate expertise. This evaluation would include an assessment of the overall disease activity, as well as a description of specific histopathological features of the disease. In CL08, optional liver biopsies for evaluation of hepatic histology were obtained at weeks 48, 96, and 144. As in the VITAL study, liver biopsies were performed with consent from the patient’s parent or legal guardian and where local regulations permitted; were subject to discretionary approval from each center’s institutional review board/independent ethics committee; and were to be performed according to the local institutional practices by a qualified professional. Sites were provided with a histopathology manual detailing the procedures for collecting and processing the liver biopsy specimens. All biopsies were centrally evaluated by an independent expert hepatopathologist from Inova Fairfax Hospital (Falls Church, VA, US). Liver histopathology was largely based on a thorough examination of sections stained with hematoxylin and eosin:

- Percent steatosis by morphometry
- Portal inflammation (scored 0–4), where 0 = none; 1 = mild, some or all portal areas; 2 = moderate, some or all portal areas; 3 = moderate/marked, all portal areas; 4 = marked, all portal areas
- Lobular inflammation (scored 0–4), where 0 = none; 1 = one focus or less per 10X objective; 2 = two to four foci per 10X objective; 3 = five to ten foci per 10X objective; 4 = more than ten foci per 10X objective
- Microvesicular steatosis and macrovesicular steatosis (scored 0–4), where 0 = none; 1 = fat vacuoles replacing less than 5% of the hepatocyte area; 2 = fat vacuoles replacing 5% to 33% of hepatocyte area; 3 = fat vacuoles replacing 33% to 66% of hepatocyte area; 4 = fat vacuoles replacing more than 66% of hepatocyte area

Additional stains were used to identify features not easily seen on a hematoxylin and eosin stain:

- Ishak stage (scored 0–6) (Sirius Red stain), where stage 0 = no fibrosis (normal); stage 1 = portal fibrosis (mild); stage 2 = portal fibrosis (moderate to severe); stage 3 = bridging fibrosis (few bridges); stage 4 = bridging fibrosis (many bridges); stage 5 = early cirrhosis; stage 6 = established or advanced cirrhosis
- Percent collagen (Sirius Red stain)
- Percent macrophages (CD68 stain)
- Percent fibrogenic cells (smooth muscle actin stain)

Changes from baseline in hemoglobin levels were measured in both studies. The proportion of patients achieving TFHN for at least 4 weeks at any time (short-term TFHN) and the proportion of patients who maintained TFHN for at least 13 weeks beginning at week 6 (sustained early TFHN) were calculated.

Lipid parameters were exploratory efficacy measures assessed at baseline and over time in both studies, and included low-density lipoprotein cholesterol, high-density lipoprotein cholesterol, total cholesterol, and triglyceride levels.

#### Nutritional status

Dietary changes, including discontinuation of a low-fat/low-cholesterol diet and/or introduction of an unrestricted, age-appropriate diet, were monitored in both studies.

### Safety assessments

Major safety outcomes in both studies included discontinuations, TEAEs, SAEs including IARs, laboratory results, vital signs, physical examination results, and development of ADAs. The severity of TEAEs was graded according to the National Cancer Institute Common Terminology Criteria for Adverse Events, version 4.0 (VITAL) or Clinical Data Interchange Standards Consortium Study Data Tabulation Model standard terminology, version 3.1.1 (CL08), and all adverse events were coded using the Medical Dictionary for Regulatory Activities, version 20.1. As previously reported, in VITAL, clinical laboratory tests were analyzed by local laboratories [9]. In CL08, all clinical laboratory tests, with the exception of coagulation parameters, were analyzed by a central laboratory (Covance; Indianapolis, IN, US; Geneva, Switzerland; or Singapore); coagulation parameters were analyzed by the site’s local laboratory. Assessment of results as normal or abnormal was based on age- and gender-specific normal ranges provided by the central or local laboratory at the time of the test. In both studies, ADAs and neutralizing antibodies in serum were analyzed by a central laboratory (Covance; Chantilly, VA, US). ADAs were detected and confirmed using an enzyme-linked immunosorbent assay (ELISA); a patient was considered to be ADA-positive if a positive result was obtained on both the screening and confirmatory ELISAs [9]. ADA titers were determined for all ADA-positive patients by serial dilution and were reported as the dilution yielding an optical density response at the ADA assay cut point. Patients with a confirmed ADA-positive result were also tested for the presence of neutralizing antibodies that inhibited sebelipase alfa enzyme activity and/or cellular uptake. *LIPA* mutation analysis was performed by Prevention Genetics (Marshfield, WI, US); functional effects of identified sequence changes were assessed using PolyPhen-2, SIFT, and MutationTaster. In CL08, the presence of whole *LIPA* deletions was determined based on testing performed at a local laboratory.

### Statistical analyses

The full analysis set included all patients who received at least 1 infusion of sebelipase alfa. In VITAL, the proportion of patients surviving to 12 months of age was calculated, along with an exact 95% CI based on the Clopper-Pearson method; the proportion of patients surviving to 18 and 24 months of age was estimated by Kaplan–Meier (product-limit) methodology. In CL08, survival was analyzed as the proportion of patients surviving to 12, 18, 24, and 36 months of age, along with an exact 95% CI based on the Clopper-Pearson method. In addition, a Kaplan–Meier estimate and exact 95% CI for median survival were determined and a Kaplan–Meier estimate and 95% CI for median time to short-term TFHN were calculated. Given the extreme rarity of this disease, the planned enrollment was approximately 8 to 10 patients in each study; no formal sample size calculations were performed. The studies were not powered to make any statistical inferences.

## Supplementary information

**Additional file 1:** Protocol-Defined Dose Escalation Criteria.

## Data Availability

Alexion will consider requests for disclosure of clinical study participant-level data provided that participant privacy is assured through methods like data de-identification, pseudonymization, or anonymization (as required by applicable law), and if such disclosure was included in the relevant study informed consent form or similar documentation. Qualified academic investigators may request participant-level clinical data and supporting documents (the statistical analysis plan and protocol) for Alexion-sponsored studies. Further details regarding data availability and instructions for requesting information are available in the Alexion Clinical Trials Disclosure and Transparency Policy at https://alexion.com/our-research/research-and-development. Link to data request form: https://alexion.com/contact-alexion/medical-information.

## Abbreviations

ADA: Anti-drug antibody
ALT: Alanine aminotransferase
AST: Aspartate aminotransferase
CI: Confidence interval
ELISA: Enzyme-linked immunosorbent assay
HSCT: Hematopoietic stem cell transplantation
IAR: Infusion-associated reaction
IV: Intravenous
LAL: Lysosomal acid lipase
LAL-D: Lysosomal acid lipase deficiency
qow: Every other week
qw: Once weekly
SAE: Serious adverse event
TEAE: Treatment-emergent adverse event
TFHN: Transfusion-free hemoglobin normalization
UK: United Kingdom
US: United States
VITAL: Survival of LAL-D Infants Treated With Sebelipase Alfa
WHO: World Health Organization

## References

[1] Grabowski GA, Valayannopoulos V, Goodman ZD, Balwani M, Valle D, Beaudet AL, Vogelstein B, Kinzler KW, Antonarakis SE, Ballabio A (2019). Lysosomal acid lipase deficiency: the continuous spectra of disease variants. The Online Metabolic and Molecular Bases of Inherited Disease.

[2] Burton B, Balwani M, Feillet F, Baric I, Burrow T, Camarena Grande C (2015). A phase 3 trial of sebelipase alfa in lysosomal acid lipase deficiency. N Engl J Med.

[3] Reiner Z, Guardamagna O, Nair D, Soran H, Hovingh K, Bertolini S (2014). Lysosomal acid lipase deficiency: an under-recognized cause of dyslipidaemia and liver dysfunction. Atherosclerosis.

[4] Rader DJ (2015). Lysosomal acid lipase deficiency - a new therapy for a genetic lipid disease. N Engl J Med.

[5] Burton BK, Deegan PB, Enns GM, Guardamanga O, Simon H, Hovingh GK (2015). Clinical features of lysosomal acid lipase deficiency: a longitudinal assessment of 48 children and adults. J Pediatr Gastroenterol Nutr.

[6] Hamilton J, Jones I, Srivastava R, Galloway P (2012). A new method for the measurement of lysosomal acid lipase in dried blood spots using the inhibitor Lalistat 2. Clin Chim Acta.

[7] Del Angel G, Hutchinson AT, Jain NK, Forbes CD, Reynders J (2019). Large-scale functional LIPA variant characterization to improve birth prevalence estimates of lysosomal acid lipase deficiency. Hum Mutat.

[8] Jones SA, Banikazemi M, Bialer M, Cederbaum S, Chan A, Dhawan A (2016). Rapid progression and mortality of lysosomal acid lipase deficiency presenting in infants. Genet Med.

[9] Jones SA, Rojas-Caro S, Quinn AG, Friedman M, Marulkar S, Ezgu F (2017). Survival in infants treated with sebelipase alfa for lysosomal acid lipase deficiency: an open-label, multicenter, dose-escalation study. Orphanet J Rare Dis.

[10] Mayatepek E, Seedorf U, Wiebusch H, Lenhartz H, Assmann G (1999). Fatal genetic defect causing Wolman disease. J Inherit Metab Dis.

[11] Anderson RA, Bryson GM, Parks JS (1999). Lysosomal acid lipase mutations that determine phenotype in Wolman and cholesterol ester storage disease. Mol Genet Metab.

[12] Wolman M (1995). Wolman disease and its treatment. Clin Pediatr (Phila ).

[13] Kanuma^®^ (sebelipase alfa) [package insert]. New Haven, CT: Alexion Pharmaceuticals Inc.; December 2015.

[14] Tracking Progress on Child and Maternal Nutrition (2009). A survival and development priority.

[15] Ghosh A, Petts G, Batra G, Wynn RF, Brenchley P, Kaur A, et al. Membranous nephropathy in a patient with infantile onset lysosomal acid lipase deficiency and anti-sebelipase antibodies [poster]. WORLD Symposium Annual Meeting on Lysosomal Disease Research; 2019 February 4–8, 2019; Orlando, FL.

[16] Huffaker MF, Liu AY, Enns GM, Vijay S, Amor AJ, Adkinson NF (2019). Case series of sebelipase alfa hypersensitivity reactions and successful sebelipase alfa rapid desensitization. JIMD Rep.

[17] Burton BK, Whiteman DA (2011). Incidence and timing of infusion-related reactions in patients with mucopolysaccharidosis type II (Hunter syndrome) on idursulfase therapy in the real-world setting: a perspective from the Hunter Outcome Survey (HOS). Mol Genet Metab.

[18] WHO Child Growth Standards (2006). Length/height-for-age, weight-for-age, weight-for-length, weight-for-height and body mass index-for-age: methods and development.

[19] WHO child Growth Standards (2007). Head circumference-for-age, arm circumference-for-age, triceps skinfold-for-age and subscapular skinfold-for-age: methods and development.

[20] Kuczmarski RJ, Ogden CL, Guo SS, Grummer-Strawn LM, Flegal KM, Mei Z (2000). CDC growth charts for the United States: methods and development. Vital Health Stat.

[21] Frankenburg WK, Dodds J, Archer P, Shapiro H, Bresnick B (1992). The Denver II: a major revision and restandardization of the Denver Developmental Screening Test. Pediatrics.

