# Correction to: Long-term survival with sebelipase alfa enzyme replacement therapy in infants with rapidly progressive lysosomal acid lipase deficiency: final results from 2 open-label studies

**DOI:** 10.1186/s13023-021-01753-0

**Published:** 2021-03-01

**Authors:** Suresh Vijay, Anais Brassier, Arunabha Ghosh, Simona Fecarotta, Florian Abel, Sachin Marulkar, Simon A. Jones

**Affiliations:** 1 Birmingham Children’s Hospital NHS Foundation Trust, Steelhouse Lane, Birmingham, B4 6NH UK; 2 Hospital Necker Enfants Malades, APHP, Paris, France; 3 Manchester Centre for Genomic Medicine, Manchester University NHS Foundation Trust, St. Mary’s Hospital, Manchester, UK; 4 Federico II University, Naples, Italy; 5 Alexion Pharmaceuticals, Inc., Boston, MA USA

## Correction to: Orphanet J Rare Dis (2021) 16:13 https://doi.org/10.1186/s13023-020-01577-4

Following the publication of the original article [1], we were informed that the Plain Language Summary had inadvertently been omitted during typesetting.

The Plain Language Summary is shown here below and has already been added back to the original article.

## Plain Language Summary

- Lysosomal acid lipase deficiency (LAL-D) is a rare, inherited disease in which fatty material (cholesterol and triglycerides) becomes trapped in cells throughout the body, causing organ damage
- Infants can experience a particularly aggressive form of this disease where the functioning of the liver and intestine is impaired, thus leading to an enlarged abdomen and failure to grow and thrive
- If left untreated, LAL-D in infants leads to death, usually by 6 months of age
- This publication reports the results from 2 studies involving 19 infants with rapidly progressive LAL-D; infants received once-weekly intravenous infusions of sebelipase alfa for up to 3 or 5 years, depending on the study
- Results show that with sebelipase alfa treatment, the likelihood of an infant with LAL-D surviving to 12 months of age is 79% and the likelihood of surviving to 5 years of age is 68%
- Throughout both studies, treatment with sebelipase alfa was associated with (1) improvements in growth (weight, length/height, and arm circumference), (2) improvements in liver function, and (3) a decrease in liver and spleen size
- All patients experienced 1 or more adverse events (unwanted side effects), most of which were mild or moderate in severity; no patient stopped receiving treatment because of these events
