# Supplementary material for: Long-term survival with sebelipase alfa enzyme replacement therapy in infants with rapidly progressive lysosomal acid lipase deficiency: final results from 2 open-label studies
Source: Orphanet J Rare Dis. 2021 Jan 6;16:13. doi: 10.1186/s13023-020-01577-4 (PMC7789691; doi:10.1186/s13023-020-01577-4)
Supplement: Supplementary file 1 — Additional file 1: Protocol-Defined Dose Escalation Criteria. [file 13023_2020_1577_MOESM1_ESM.docx]

**Long-Term Survival With** **Sebelipase Alfa Enzyme Replacement Therapy in**

**Infants With Rapidly Progressive Lysosomal Acid Lipase Deficiency:**

**Final Results From 2 Open-Label Studies**

Suresh Vijay, Anais Brassier, Arunabha Ghosh, Simona Fecarotta, Florian Abel, Sachin Marulkar, Simon A. Jones

**File name: Vijay et al_Additional File 1 for Submission_9-21-20.doc**

**Title: SUPPLEMENTARY APPENDIX**

**Description of data: Protocol-Defined Dose Escalation Criteria**

**SUPPLEMENTARY APPENDIX**

**Protocol-Defined Dose Escalation Criteria**

*VITAL*

Prior to considering a dose increase, the patient was to be evaluated for other potential causes of any observed clinical manifestations, which could include missed study infusions, onset of acute cholecystitis or initiation of a potentially hepatotoxic concomitant medication in a patient with elevated alanine aminotransferase (ALT), or concomitant illness in a patient with reduced weight gain, such as an upper respiratory tract infection or other childhood viral infection.

Within a patient's first 3 months of treatment, the patient could be considered for dose escalation to 3.0 mg/kg once weekly (qw) if the patient met 2 or more of the following criteria after receiving at least 4 infusions at a dose of 1.0 mg/kg qw:

- Failure to gain an average of 5 g/kg body weight per day AND the presence of either of the following:
  - World Health Organization (WHO) weight-for-length or weight-for-height *z* score less than −2
  - WHO length-for-age or height-for-age *z* score less than −2
- Albumin less than 3.5 g/dL
- ALT more than twice the upper limit of normal
- Ongoing requirement for blood and/or platelet transfusion

After 3 months of treatment, a patient could be considered for dose escalation from 1.0 mg/kg qw to 3.0 mg/kg qw if the patient had any other clinically important manifestation of LAL-D on clinical examination, laboratory assessment, or imaging that had either not improved from a minimum of 3 assessments, or had failed to normalize within 12 months of treatment. Such manifestations included but were not restricted to a decrease in weight-for-age crossing at least 2 major centiles, serum transaminase or albumin levels meeting the above criteria, or the presence of hepatomegaly, splenomegaly, or lymphadenopathy.

During the conduct of the study, the protocol was amended to include an option for patients who continued to meet dose-escalation criteria after receiving at least 4 infusions at a dose of 3.0 mg/kg qw to be considered for a dose escalation to 5.0 mg/kg qw on a case-by-case basis. The dose-escalation criteria were those previously defined for patients who had been on treatment for at least 3 months.

*CL08*

Prior to considering a dose increase, the patient was to be evaluated for other potential causes of any observed clinical manifestations, which could include missed study infusions, initiation of a potentially hepatotoxic concomitant medication in a patient with elevated ALT, or concomitant illness in a patient with reduced weight gain, such as an upper respiratory tract infection or other childhood viral infection.

Within the first 3 months of treatment, a patient could be considered for dose escalation to 3.0 mg/kg qw if the patient met 2 or more of the following criteria while receiving a dose of 1.0 mg/kg qw:

- Failure to gain an average of 5 g/kg body weight per day and the presence of 1 or more of the following:
  - WHO weight-for-length or weight-for-height *z* score less than −2
  - WHO length-for-age or height-for-age *z* score less than −2
  - WHO weight-for-age *z* score less than −2
- Albumin less than 3.5 g/dL
- ALT more than twice the upper limit of normal
- Ongoing requirement for blood and/or platelet transfusion

After 3 months of treatment, a patient could be considered for dose escalation to 3.0 mg/kg qw if the patient met at least 1 of the criteria outlined above, or had any other clinically important manifestation of LAL-D on clinical examination, laboratory assessment, or imaging that had not improved from baseline, had improved and plateaued but had not normalized (where plateauing is considered a minimum of 3 assessments), or had failed to normalize within 12 months of treatment.

During the conduct of the study, the protocol was amended to include an option for dose escalation to 5.0 mg/kg qw. A patient could be considered for a dose escalation to 5.0 mg/kg qw if the patient met the dose-escalation criteria defined above after receiving at least 4 infusions at a dose of 3.0 mg/kg qw. In rare circumstances, dose escalation to 5.0 mg/kg qw could be considered prior to 4 infusions, if approved by the sponsor and the Safety Committee.

Under a subsequent country-specific protocol amendment, a patient receiving treatment in the UK could be considered for a dose escalation to 7.5 mg/kg qw if a thorough case review indicated that a patient continued to have evidence of disease progression at a dose of 5.0 mg/kg qw, as judged based on the criteria referenced above, as well as other manifestations of disease progression (eg, recurrent infections, poor feeding, and vomiting).
